# Psychopathology of addiction: May a SCL-90-based five dimensions structure be applied irrespectively of the involved drug?

**DOI:** 10.1186/s12991-016-0100-8

**Published:** 2016-04-26

**Authors:** Pier Paolo Pani, Angelo G. I. Maremmani, Emanuela Trogu, Federica Vigna-Taglianti, Federica Mathis, Roberto Diecidue, Ursula Kirchmayer, Laura Amato, Joli Ghibaudi, Antonella Camposeragna, Alessio Saponaro, Marina Davoli, Fabrizio Faggiano, Icro Maremmani

**Affiliations:** Social and Health Services, Cagliari Public Health Trust (ASL Cagliari), Cagliari, Italy; Piedmont Centre for Drug Addiction Epidemiology, ASLTO3, Grugliasco, Province of Turin Italy; Department of Clinical and Biological Sciences, San Luigi Gonzaga University, Turin, Regione Gonzole 10, 10043 Orbassano, Province of Turin Italy; Department of Psychiatry, Cagliari Public Health Trust (ASL Cagliari), Cagliari, Italy; Department of Epidemiology, Latium Regional Health Service, Rome, Italy; National Coordination Hospitality Communities (CNCA), Rome, Italy; Regional Epidemiological Observatory, Emilia Romagna Regional Health Service, Bologna, Italy; Department of Translational Medicine, Avogadro University, Novara, Italy; Vincent P. Dole Dual Diagnosis Unit, Department of Neurosciences, Santa Chiara University Hospital, University of Pisa, Via Roma, 67, 56100 Pisa, Italy; Association for the Application of Neuroscientific Knowledge to Social Aims (AU-CNS), Pietrasanta, Lucca Italy; G. De Lisio Institute of Behavioural Sciences, Pisa, Italy

**Keywords:** Psychopathology, Addiction, Heroin, Alcohol, Cocaine, SCL-90

## Abstract

**Background:**

We previously found a five cluster of psychological symptoms in heroin use disorder (HUD) patients: ‘worthlessness-being trapped’, ‘somatic-symptoms’, ‘sensitivity-psychoticism’, ‘panic-anxiety’, and ‘violence-suicide’. We demonstrated that this aggregation is independent of the chosen treatment, of intoxication status and of the presence of psychiatric problems.

**Methods:**

2314 Subjects, with alcohol, heroin or cocaine dependence were assigned to one of the five clusters. Differences between patients dependent on alcohol, heroin and cocaine in the frequency of the five clusters and in their severity were analysed. The association between the secondary abuse of alcohol and cocaine and the five clusters was also considered in the subsample of HUD patients.

**Results:**

We confirmed a positive association of the ‘somatic symptoms’ dimension with the condition of heroin versus cocaine dependence and of the ‘sensitivity-psychoticism’ dimension with the condition of alcohol versus heroin dependence. ‘Somatic symptoms’ and ‘panic anxiety’ successfully discriminated between patients as being alcohol, heroin or cocaine dependents. Looking at the subsample of heroin dependents, no significant differences were observed.

**Conclusions:**

The available evidence coming from our results, taken as a whole, seems to support the extension of the psychopathological structure previously observed in opioid addicts to the population of alcohol and cocaine dependents.

## Background

The relationship between substance use and other psychiatric disorders is still an open question. According to the current nosographic approach, substance use disorders are defined by the presence of the core symptoms of addiction, such as continuation of use despite consequences, reduction of other interests and activities, craving, dyscontrol, and so on. Other distinctive psychopathological symptoms of people with addictive behaviours are included in separate disorders pertaining to the domain of psychiatric ‘comorbidity’. Several findings challenge this fragmentary approach to unitary clinical presentations and call for a revision of the present nosology. Besides the high degree of association between core symptoms of addiction and symptoms of other psychiatric diseases [1–3], further neurobiological and clinical considerations highlight the strong sharing of features between addiction per se and other psychopathological disorders, especially in the mood, anxiety and impulse/control domains [4]. On these bases, a unitary perspective in the approach to patients with addiction, including psychological and psychiatric vulnerability, the effect of substances, the characteristics of addictive processes, and their interactions has been proposed [4].

In view of the persistent uncertainty in classifying psychiatric symptomatology as intrinsic to the addictive disorder or due to psychiatric comorbidity, a low level of inference on the psychopathology of addicted people has been applied recently, so that the symptoms expressed by patients are examined independently of a pre-established syndromic level [such as that of the Diagnostic and Statistical Manual of Mental Disorders (DSM)]. In a previous study that took this approach, we investigated the psychopathological dimensions of 1055 heroin addicts entering opioid agonist treatment (OAT) [5]. By applying an exploratory principal component factor analysis (PCA) to the 90 items on the SCL-90 checklist, a 5-factor solution was identified: the first factor reflected a depressive ‘worthlessness and being trapped’ dimension; the second factor picked out a “somatic symptoms” dimension; the third identified a ‘sensitivity-psychoticism’ dimension; the fourth a ‘panic anxiety’ dimension; and the fifth a ‘violence-suicide’ dimension. These same results were recently replicated by applying the PCA to another Italian sample of 1195 heroin addicts entering a Therapeutic Community Treatment [6]. Although this second sample differed from the first one in important sociodemographic and clinical factors, treatment setting, and programme characteristics, the same five psychopathological dimensions were found, suggesting the existence of specific clusters of psychological/psychiatric features within the category of opioid addicts.

Further analyses confirmed the clusters of symptoms, independently of demographic and clinical characteristics, active heroin use, lifetime psychiatric problems, and kind of treatment received by the patient [6–8]. The available evidence, taken as a whole, seems to support the trait dependent, rather than the state dependent, nature of the proposed factorial dimensions of the psychopathology of opioid addiction.

Our previous considerations on the nature of the relationship between addiction and other psychiatric domains, corroborated by neurobiological, epidemiological, and clinical evidence do not apply to heroin dependence only, but also to the other addictive disorders, which suggests that the investigation should be extended to other addictions.

The primary aim of the present study is to investigate whether the five-factor psychic structure found in opioid dependents also applies to cocaine- or alcohol-dependent patients. A secondary aim is to evaluate the impact of the use of other substances on the five cited psychopathological dimensions. On the basis of our unitary hypothesis, we expect that the use of other substances is likely to have

- A pathoplastic effect on psychological/psychiatric dimensions, related to the specific psychoactive properties of each substance;
- No substantial effect on the five-dimension structure of psychopathology, as we believe this structure is intrinsic to the condition of addiction per se and is independent of the substance.

## Methods

### Study design and population

The VOECT (Evaluation of Therapeutic Community Treatments and Outcomes) cohort study was conducted in 8 Italian regions in 2008–2009, recruiting a total of 2533 patients admitted to a Therapeutic Community (TC) treatment for a substance use disorder [9]. To fulfil the aims of the present analysis only baseline data were used, implementing a cross-sectional approach. Moreover, specific inclusion criteria were applied: at least 18 years old, with a diagnosis of heroin, cocaine or alcohol dependence based on a clinical judgement, and information available from the SCL-90 questionnaire. Reflecting these criteria, the sample consisted of 2314 subjects. Each participant was included in the sample once only. Out of 2314 patients, 449 (19.4 %) were diagnosed as dependent on alcohol, 670 (28.9 %) on cocaine, and 1195 (51.6 %) on heroin.

The study was conducted according to the WMA Declaration of Helsinki—Ethical Principles for Medical Research Involving Human Subjects. All subjects examined filled up an informed consensus to participate in this study. Both the consent form and the experimental procedures were approved by the pertinent ethics committees in accordance with internationally accepted criteria for ethical research.

### Instruments

#### Self-report symptom inventory (SCL-90)

Developed by Derogatis et al. [10], the SCL90 consists of 90 items, each rated on a 5-point scale of distress. It is a self-report clinical rating scale oriented to the collection of symptomatic behaviours of psychiatric outpatients. Among heroin-dependent patients, the 90 items reflected the 5 primary symptom dimensions which are believed to underlie the large majority of symptom behaviours observed in this kind of patient: worthlessness-being trapped, somatic symptoms, sensitivity-psychoticism, panic-anxiety, and violence-suicide [5].

The worthlessness-being trapped dimension reflects a broad range of the symptoms typical of the clinical depressive syndrome. This dimension mirrors feelings of worthlessness and of being trapped or caught. The somatic symptoms dimension reflects distress arising from perceptions of body dysfunctions. The sensitivity-psychoticism dimension focuses on feelings of a full continuum of psychotic behaviours. The panic-anxiety dimension subsumes a set of symptoms and experiences usually clinically associated with a high level of manifest anxiety. The violence-suicide dimension is organized around three categories of hostile behaviour: thoughts, feelings, and actions; it also comprises thoughts of death and suicidal ideation.

In a previous study, these five dimensions were empirically established and validated [7, 8, 11, 12]. In the present study, SCL-90 was administered to the patients within 15 days from the entry into TC.

#### Other instruments

Information on the sociodemographic and clinical characteristics of the patients included in the study was collected through a research questionnaire administered at the time of entering TC. The secondary substance of abuse (one or more) was self-reported by the patient.

### Data analysis

Two analysis sets were obtained. The principal analysis was run on the full sample of subjects entering TC treatment for opioid, cocaine, or alcohol dependence (*n* = 2314); this sample was divided into three groups according to the primary substance of abuse. The secondary analysis was performed on subjects entering a TC treatment for opioid dependence. Only opioid addicts reporting cocaine, alcohol, or neither of these as secondary substances of abuse were included (*n* = 947), while those reporting both as secondary substances of abuse were excluded. Patients were compared as regards demographic and clinical variables by means of the Chi-square test (with Bonferroni’s correction) for categorical variables, and Student’s *t* test for continuous variables.

SCL-90 factors were standardized into *z*-scores in order to make scores comparable. Each subject was assigned to one of the five subtypes on the basis of the highest *z*-score achieved (named “dominant SCL-90 factor”). This procedure gives the opportunity to classify subjects on the basis of the highest symptomatological cluster, overcoming the problem of identifying a cut-off point for the inclusion of patients in the clusters. The subtypes are clearly distinct, as shown by analysing the mean *z*-scores and 95 % confidence interval (CI 95 %) across the factors for each dominant group [5]. Psychopathological symptoms were then compared by means of the Chi-square test, with Bonferroni’s correction, between patients with primary dependence on alcohol, heroin, or cocaine; and between patients with primary dependence on opioids and secondary use of cocaine, alcohol, or neither of these substances.

Stepwise multinomial logistic regression analysis was then conducted with the primary substance of abuse (heroin, cocaine, or alcohol) as dependent variable and the psychopathological dominant groups as predictors. In our model, we included sociodemographic and clinical variables that may act as confounding factors (age, gender, marital status, detoxification status, living condition). A second logistic regression investigating cocaine versus alcohol as primary substance of abuse and including the same sociodemographic and clinical variables as had been used in the previous model as possible confounders was also carried out.

SCL-90 5-factor solution scores were compared on the base of the primary or secondary abused substance at univariate (*t* test) and multivariate (discriminant analysis) level. Discriminant analysis is useful to statistically distinguish between two or more groups of cases, and is also a powerful classification technique. By classifying the cases used to derive the functions, and comparing predicted group membership with actual group membership, one can empirically measure the success in discrimination by observing the proportion of correct classifications. The purpose is to see how effective the discriminating variables are. If a large proportion of misclassification occurs, then the selected variables are poor discriminators. We used the stepwise procedure to select the best discriminating variables.

All analyses were carried out using SPSS v. 4.0 (SPSS, Chicago, IL, USA). Statistical significance was set at the *p* = 0.05 level.

## Results

### Sociodemographic and addiction characteristics

In the overall sample (*n* = 2314), mean age was 35.2 ± 8.5 years, 84.2 % of the subjects were male, 87.9 % were single, separated, divorced, or a widow(er), 20.5 % had a high educational level (with duration over 8 years), 79.1 % of subjects were unemployed, and 26.5 % lived alone.

When looking at differences between alcohol, heroin and cocaine patients, mean age was 41.4 ± 8.8 among alcohol-dependent, 33.8 ± 7.7 among heroin-dependent, and 33.6 ± 7.7 among cocaine-dependent patients. This difference was statistically significant (*F* = 168.87; *p* = 0.000). As regards gender, the frequency of males was 85.2 % among patients with alcohol dependence, 81.4 % among those with heroin dependence, and 88.7 % among those with cocaine dependence (Chi-square = 17.30; *p* = 0.000). The frequency of single/separated/divorced/widow(er) subjects was 87.5, 90.2 and 84.1 % among alcohol-, heroin-, and cocaine-dependent patients, respectively (Chi-square = 14.88; *p* = 0.001). Educational level was high (covering over 8 years) in 20.5 % of the alcohol sample, 21.5 % of the heroin sample, and 18.7 % of the cocaine sample, without statistically significant differences between groups (Chi-square 2.1; *p* = 0.366). 77.7 % of patients with alcohol dependence, 79.8 % of those with heroin dependence, and 78.8 % of those with cocaine dependence were unemployed (Chi-square = 0.95; *p* = 0.621). 37.5, 24.1, and 23.4 % for the alcohol, heroin, and cocaine group of patients, respectively, were living alone (chi = 23.71; *p* = 0.000).

Among patients with opioid dependence (*n* = 1195), mean age was 34.45 ± 7.1 for the group with alcohol as secondary substance of abuse, 33.64 ± 7.5 for those with cocaine, and 35.61 ± 7.6 for those with neither of these substances as secondary substance of abuse (*F* = 2.91; *p* = 0.055). The frequency of males was 78.5 % among patients who used alcohol as secondary substance of abuse, 82.9 % among those who used cocaine, and 76.5 % among those who used neither of the two (Chi-square = 2.83; *p* = 0.243). The frequency of subjects who were single, separated, divorced, or a widow(er), was 88.6, 90.9, and 84.7 %, respectively (Chi-square = 3.53; *p* = 0.170). Educational level was high (covering over 8 years) in 31.6 % of those whose secondary substance of abuse was alcohol, 19.5 % with cocaine, and 28.2 % of the patients using neither as secondary substance (Chi-square = 9.06; *p* = 0.011). 81.0 % of patients whose secondary substance of abuse was alcohol, 82.5 % of those using cocaine, and 65.9 % of those using neither as secondary substance were unemployed (Chi-square = 13.65; *p* = 0.001). The percentages for those living alone were 32.9, 24.4, and 19.3 % for those using alcohol, cocaine, or neither of the two as secondary substance of abuse, respectively (chi = 4.08; *p* = 0.130).

### Psychopathological dimensions

Table 1 shows differences in psychopathological symptoms between patients with addiction to alcohol, heroin, or cocaine as primary substance of abuse. The most frequent psychopathological dimension was ‘panic-anxiety’ for all the three groups of patients. The least frequent psychopathological dimensions were ‘violence-suicide’ for the group of patients with alcohol dependence (11.4 %) and ‘worthlessness-being trapped’ for the patients with heroin or cocaine dependence (15.1 and 16.0 %, respectively).

**Table 1:** SCL90-based predominant psychopathological symptoms according to the primary substance of abuse at treatment entry

Dominant group	Total *N* = 2314	Alcohol *N* = 449	Heroin *N* = 1195	Cocaine *N* = 670
		*N* (%)	*N* (%)	*N* (%)
1. Worthlessness-being trapped	349 (15.1)	62 (13.8)a	180 (15.1)a	107 (16.0)a
2. Somatic symptoms	509 (22.0)	102 (22.7)a,b	291 (24.4)b	116 (17.3)a
3. Sensitivity-psychoticism	465 (20.1)	110 (24.5)a	218 (18.2)b	137 (20.4)a,b
4. Panic-anxiety	605 (26.1)	124 (27.6)a	300 (25.1)a	181 (27.0)a
5. Violence-suicide	386 (16.7)	51 (11.4)a	206 (17.2)b	129 (19.3)b

Chi square 28.61; *df* 8; *p* < 0.001

Each letter denotes a subset of categories whose column proportions do not differ significantly from each other at the 0.05 level

The only statistically significant differences observed between the three groups were in the frequency of

- The ‘somatic symptoms’ dimension, which was better represented in the group of patients with heroin than in the group with cocaine dependence;
- ‘Sensitivity-psychoticism’, which was more frequent in the group of patients with alcohol than in the group with heroin dependence;
- The ‘violence-suicide’ dimension, which had a higher frequency in the groups of patients with heroin or cocaine than in the group with alcohol dependence.

The multinomial logistic regression confirmed a significant positive association of the ‘somatic-symptoms’ dimension with the heroin versus the cocaine group and of the ‘sensitivity-psychoticism’ dimension with the alcohol versus the heroin group (Table 2). The further logistic regression investigating cocaine versus alcohol as primary substance of abuse did not detect any significant association between the five SCL 90 dominant groups and the primary substance of abuse.

**Table 2:** Risk factors for alcohol or cocaine versus heroin dependence at treatment entry, according to logistic regression (only significant results are reported), 2314 patients

Primary substance of abuse^a^	B	Odds ratio	95 % CI	*p*
Alcohol				
Age	0.11	1.11	1.09–1.13	0.000
Living alone	0.32	1.38	1.06–1.80	0.017
Detoxified status	0.61	1.84	1.44–2.35	0.000
SCL90 dominant groups				
Prominent sensitivity-psychoticism^b^	0.43	1.54	1.02–2.32	0.039
Cocaine				
Male gender	0.53	1.70	1.27–2.27	0.000
Single marital status	−0.53	0.59	0.43–0.80	0.001
Detoxified status	0.45	1.57	1.29–1.92	0.000
SCL90 dominant groups				
Prominent somatic symptoms^b^	−0.39	0.68	0.48–0.95	0.023

Statistics: Chi-square 375.88; *df* 20; *p* < 001

This table includes SCL-90 factors as exposure variables, other demographic and clinical variables as possible confounders, and primary substance of abuse (alcohol or cocaine vs. heroin) as a dependent variable

^a^Considering heroin as reference primary substance of abuse

^b^Considering prominent worthlessness-being trapped symptoms as reference psychopathology

Table 3 shows differences in psychopathological symptoms between patients with opioid dependence and secondary abuse of alcohol, or else cocaine or neither of these drugs. The most frequent psychopathological dimension was ‘panic-anxiety’ for all the three groups of patients The less frequent were ‘violence-suicide’ and ‘sensitivity-psychoticism’ for the group of patients with alcohol as secondary abused drug, ‘violence-suicide’ for the group of patients with no alcohol or cocaine as secondary drug, and ‘worthlessness-being trapped’ for the group of patients with cocaine as secondary abused drug. No statistically significant differences between the three groups were observed in any of the five SCL-based psychopathological dimensions.

**Table 3:** SCL90-based predominant psychopathological symptoms in heroin-dependent patients according to their secondary substance of abuse

SCL90-based factors	Secondary substance of abuse		
	Alcohol *N* = 79	Cocaine *N* = 783	Neither alcohol nor cocaine *N* = 85
	*N* (%)	*N* (%)	*N* (%)
1. Worthlessness-being trapped	15 (19.0)	113 (14.4)	12 (14.1)
2. Somatic symptoms	17 (21.5)	191 (24.4)	18 (21.2)
3. Sensitivity-psychoticism	14 (17.7)	138 (17.6)	20 (23.5)
4. Panic-anxiety	19 (24.1)	199 (25.4)	25 (29.4)
5. Violence-suicide	14 (17.7)	142 (18.1)	10 (11.8)

Chi-square = 5.38; *df* = 8; *p* = 0.715

Post-hoc test (Bonferroni’s procedure): no differences

With reference to psychopathological severity, Table 4 shows the results of the univariate and multivariate (stepwise discriminant) analysis comparing patients with dependence on alcohol, heroin, or cocaine. All SCL-90 dimensions, with the exception of ‘violence-suicide’, were higher in the group of patients with alcohol dependence than in the group with heroin dependence, and in this second group than in the group of patients with cocaine dependence. Scores for the ‘violence-suicide’ dimension were higher in patients with cocaine dependence or heroin dependence than in those with alcohol dependence. Statistically significant differences were found for

**Table 4:** SCL90-based psychopathological factor severity in 2314 drug addicts according to the primary substance of abuse at treatment entry

Variables	Alcohol *N* = 449	Heroin *N* = 1195	Cocaine *N* = 670	Univariate *F*; df; *p*	*df* ^a^
	M ± SD	M ± SD	M ± SD		
1. Worthlessness-being trapped	1.17 ± 0.7	1.14 ± 0.8	1.11 ± 0.7	0.73; 2; 0.481	0.16
2. Somatic-symptoms	1.02 ± 0.7a	1.00 ± 0.7a	0.88 ± 0.7b	7.21; 2; 0.001	0.52
3. Sensitivity-psychoticism	0.82 ± 0.6	0.77 ± 0.6	0.76 ± 0.6	1.10; 2; 0.330	0.17
4. Panic-anxiety	0.47 ± 0.5a	0.43 ± 0.6a,b	0.38 ± 0.5b	3.72; 2; 0.024	0.38
5. Violence-suicide	0.75 ± 0.6	0.79 ± 0.7	0.80 ± 0.7	0.85; 2; 0.425	−0.16
Centroids	0.191	0.047	−0.211		

Wilks Lambda 0.97; Chi-square 55.70; *df* 10; *p* < 0.001

Correctly classified: 35.7 %

Each letter denotes a subset of categories whose column proportions do not differ significantly from each other at the 0.05 level

^a^Discriminant function (structured matrix)

- ‘Somatic symptoms’, where patients with alcohol or heroin dependence showed higher scores than patients with cocaine dependence;
- ‘Panic-anxiety, where patients with alcohol dependence showed higher scores than patients with cocaine or heroin dependence.

The stepwise analysis identified the severity of ‘somatic symptoms’ and ‘panic anxiety’ as the factors best discriminating between the three groups of patients. However, according to this analysis, the percentage of the originally grouped cases correctly classified is only 35.7 %. The other SCL-90-based psychopathological dimensions were unable to improve the value of the discrimination.

Looking at the 5-factor solution SCL-90 scores, Table 5 shows univariate and multivariate (stepwise discriminant) analysis for the sample of patients with opioid dependence and secondary abuse of alcohol, cocaine, or neither of these drugs. All the SCL-90 dimensions were higher in patients who were co-abusing alcohol or cocaine compared with those not adding these other drugs. In any case, none of the differences observed reached statistical significance and none of the five psychopathological dimensions showed a discriminant value.

**Table 5:** SCL90-based psychopathological factor severity in heroin use disorder according to the secondary substance of abuse

SCL90-based factors	Heroin + alcohol *N* = 79	Heroin + cocaine *N* = 783	Heroin (neither alcohol nor cocaine) *N* = 85	*F*	*p*
	M ± SD	M ± SD	M ± SD		
1. Worthlessness-being trapped	1.13 ± 0.7	1.15 ± 0.8	1.02 ± 0.8	1.01	0.362
2. Somatic symptoms	0.99 ± 0.7	1.02 ± 0.7	0.91 ± 0.7	0.81	0.444
3. Sensitivity-psychoticism	0.70 ± 0.5	0.81 ± 0.7	0.66 ± 0.6	2.28	0.102
4. Panic-anxiety	0.41 ± 0.5	0.45 ± 0.6	0.34 ± 0.5	1.30	0.271
5. Violence-suicide	0.69 ± 0.5	0.83 ± 0.7	0.64 ± 0.6	3.72	0.024

Post-hoc test (Scheffe’s procedure): no differences between groups in any psychopathological factor

Discriminant analysis

FD1 Wilks lambda = 0.98; Chi-square = 13.28; *df* 10, *p* = 0.208

FD2 Wilks lambda = 0.99; Chi-square = 1.26; *df* 4, *p* = 0.867

## Discussion

Patients with alcohol, heroin, or cocaine dependence differ in most of the demographic characteristics investigated in our study. Differences were observed in age, frequency of male gender, marital status, and living conditions. Alcohol dependents were older and a higher percentage of these subjects lived alone than in the case of heroin or cocaine dependents; heroin dependents were less frequently male and more frequently single than cocaine-dependent patients. In patients with primary opioid dependence, those with cocaine as secondary substance of abuse had received less education than those with alcohol as secondary substance of abuse and they were more frequently unemployed than those who had no secondary substance of abuse. These differences are consistent with the sociodemographic characteristics of the diverse populations of addicts reported in previous studies [13–15].

Looking now at the predominant psychopathological symptoms of the full sample of patients according to the primary substance of abuse at treatment entry, few significant differences were found. Taking into account sociodemographic and clinical confounding factors, the only significant difference was found in the ‘somatic symptoms’ dimension, which had a higher frequency in the heroin versus the cocaine group of patients and in the ‘sensitivity-psychotic’ dimension, which was more frequent in the alcohol versus the heroin group of patients. The higher allocation of heroin-dependent versus cocaine-dependent patients to the ‘somatic symptoms’ group may be explained by the pre-eminence of the withdrawal symptomatology in the everyday life of heroin-dependent as opposed to cocaine-dependent patients [5, 16]. The higher allocation of patients dependent on alcohol versus those dependent on heroin to the ‘sensitivity-psychotic’ dimension may be explained by the pre-eminence of the psychotic component of alcohol withdrawal as well as by the likely association of alcohol dependence, when compared with heroin dependence, including the presence of overt psychotic disorders [17–22].

Turning now to the frequency of the five predominant psychopathological dimensions in patients with primary opioid dependence, the use of alcohol or cocaine as secondary substance of abuse does not lead to any significant difference; thus the use of these additional substances appears to have no impact on these dimensions.

As regards psychiatric severity, considering the full sample, the SCL-90 average scores seem to follow a decreasing order in four of the five psychopathological dimensions, with patients who have primary alcohol dependence showing the highest and those with cocaine dependence the lowest severity. However, significant differences between the three groups were observed for the ‘somatic symptoms’ and the ‘panic-anxiety’ dimensions only, with alcohol or heroin dependence associated with higher scores for ‘somatic symptoms’ than in the case of cocaine dependence, and with alcohol dependence associated with higher scores for the ‘panic-anxiety’ dimension than in the case of cocaine dependence. Consistently with the foregoing, when discriminant analysis was applied, these two dimensions were the only factors that were able to distinguish between substance-defined groups of patients, although the discriminant power of these dimensions was rather weak, with only 35.7 % of these cases, as originally grouped, proving to be correctly classified.

The discriminating feature of the ‘somatic symptoms’ dimension may be explained by the higher burden of physical concerns that accompanies heroin or alcohol dependence, compared with dependence on cocaine, especially when considering the importance of withdrawal-related physical symptoms in heroin and alcohol dependence. Indeed, the SCL-90 items included in the ‘somatic-symptoms’ dimension correspond to a number of complaints (in particular, muscle aches, back pain, hot flushes and cold shivers, nausea, and disturbed sleep) which are usually prominent in opioid or alcohol withdrawal [5, 16]. Conversely, in cocaine dependence, withdrawal is frequently associated with mild or no somatic symptoms.

It appears to be more difficult to explain the higher severity induced by alcohol dependence than by cocaine dependence with reference to the ‘panic-anxiety’ dimension. Both forms of dependence, on alcohol and on cocaine, have been associated with a strikingly high odds ratio for the presence of anxiety disorders, and anxiety has been identified as a determinant of the progression from use of substances to dependence [1, 23, 24]. However, anxiety is a common symptom of the effects of cocaine and of cocaine intoxication, whereas in alcohol, dependence symptoms of the anxiety spectrum are expressed instead as a component of withdrawal where, even in mild forms of alcohol dependence, anxiety appears as an essential element. Moreover, we have also to take into account that alcohol withdrawal-related anxiety, unlike most of the other physical symptoms accompanying withdrawal, may last for months [25, 26]. Persistent changes in the GABA and NMDA circuits associated with tolerance and withdrawal may support the persistence of anxiety-related symptoms [27, 28]. Therefore, the highest scores observed in patients belonging to the alcohol-dependents group, as well as the contribution of the ‘panic-anxiety’ dimension to the discrimination between dependence to different substances, may reflect the weight of withdrawal-related anxiety in the everyday life of patients affected by different forms of drug addiction.

Looking at the analyses carried out with heroin-dependent patients only, no difference was shown on the basis of the use of alcohol or cocaine as secondary substance of abuse, either in the frequency of the five psychopathological dimensions or in the severity of SCL-90 scores.

Considering as a whole the results obtained by our study, it seems that when the five-factor SCL-based cluster of symptoms obtained with a population of heroin-dependent patients is compared with a different population of cocaine or alcohol dependents, only slight differences are observed, a result that can be explained by the impact of different substances on specific psychological/psychiatric dimensions. One of these dimensions—somatic symptoms—proved to be the strongest discriminating factor between the three populations considered. Even so, the best discriminating solution, which also included the panic-anxiety dimension, was only able to correctly classify 35.7 % of cases. This low discriminating power of the five SCL-90-based dimensions may be attributed to a psychopathological structure of drug addiction that is shared by the various forms of dependence, so that the structure is largely independent of the specific drug used; this result allows to define addiction as a unitary condition.

## Limitations

In this study, we have found evidence of the similarity of the SCL-90-based five-factor psychopathological structure in the three cases of heroin, cocaine, and alcohol dependence, but, given the cross-sectional design of the study, we still do not know whether the previous presence and severity of symptoms included in the five psychopathological dimensions may be a predictor of or predispose to dependence on specific substances, or whether, conversely, the use of, or dependence on these substances may condition the appearance of the specific psychopathological picture. Moreover, although we did take into account sociodemographic and clinical variables as confounding factors, other primary potential interfering factors, such as formal psychiatric diagnoses, were unavailable and could not be taken into account.

Other limits to the validity of the SCL-90-based psychopathological 5-dimension solution were commented on in our previous studies on the SCL-90 defined structure of the psychopathology of opioid addiction [5, 8, 11]. Among these, there is the lack of any observer-related ‘objective’ evaluation, as SCL-90 is a self-administered instrument that may be affected by the voluntary or involuntary hiding of some symptoms.

Lastly, a further limitation is that in this analysis we considered only the SCL-90 questionnaire that was administered at entry into treatment, so our results can only be considered to be representative of subjects who had an addiction at that particular moment. Some symptoms may vary at different stages of the disease, so that they may prove to be underweighted or overweighted in our sample.

## Conclusions

In our study, the frequencies of the five clusters of symptoms resulting from the application of PCA to the SCL-90 responses of opioid addicts turn out to differ, in a statistically significant way, only in the case of the ‘somatic symptoms’ dimension between cocaine and opioid-dependent patients and with the ‘sensitivity-psychoticism’ dimension between alcohol- and opioid-dependent subjects. Moreover, the ‘somatic symptoms’ and ‘panic-anxiety’ dimensions seem to be the only psychopathologically discriminant factors between the three populations. Looking only at the population of opioid addicts, no differences in the psychic structure were shown on the basis of the co-abuse of alcohol or cocaine.

These findings should be added to those of previous studies that had shown the considerable stability of the five-factor structure of opioid addiction. At the same time, our latest results support its extension to the population of alcohol and cocaine dependents, where the role played by the different substances seems to be that of having a pathoplastic effect on some specific dimensions, without substantially interfering with the proposed five-dimension structure, since this structure belongs to the condition of addiction per se and is independent of the various substances considered.
